# Power and sample size calculation for stepped-wedge designs with discrete outcomes

**DOI:** 10.1186/s13063-021-05542-9

**Published:** 2021-09-06

**Authors:** Fan Xia, James P. Hughes, Emily C. Voldal, Patrick J. Heagerty

**Affiliations:** 1 National Alzheimer’s Coordinating Center, University of Washington, Seattle, WA, USA; 2 Department of Biostatistics, University of Washington, Seattle, WA, USA

**Keywords:** Stepped-wedge designs, Power calculation, Non-normal outcomes, Minimal computational power

## Abstract

**Background:**

Stepped-wedge designs (SWD) are increasingly used to evaluate the impact of changes to the process of care within health care systems. However, to generate definitive evidence, a correct sample size calculation is crucial to ensure such studies are properly powered. The seminal work of Hussey and Hughes (Contemp Clin Trials 28(2):182–91, 2004) provides an analytical formula for power calculations with normal outcomes using a linear model and simple random effects. However, minimal development and evaluation have been done for power calculation with non-normal outcomes on their natural scale (e.g., logit, log). For example, binary endpoints are common, and logistic regression is the natural multilevel model for such clustered data.

**Methods:**

We propose a power calculation formula for SWD with either normal or non-normal outcomes in the context of generalized linear mixed models by adopting the Laplace approximation detailed in Breslow and Clayton (J Am Stat Assoc 88(421):9–25, 1993) to obtain the covariance matrix of the estimated parameters.

**Results:**

We compare the performance of our proposed method with simulation-based sample size calculation and demonstrate its use on a study of patient-delivered partner therapy for STI treatment and a study that assesses the impact of providing additional benchmark prevalence information in a radiologic imaging report. To facilitate adoption of our methods we also provide a function embedded in the R package “swCRTdesign” for sample size and power calculation for multilevel stepped-wedge designs.

**Conclusions:**

Our method requires minimal computational power. Therefore, the proposed procedure facilitates rapid dynamic updates of sample size calculations and can be used to explore a wide range of design options or assumptions.

## Background

Great progress has been made in public health and medical care during the past century through immunization, food safety, improvements in maternal and infant health, and advances in drugs, devices, and strategies to treat disease. However, continuing efforts to improve the quality and efficiency of care require rigorous evaluation to further guide decision-making. To evaluate novel strategies within health care delivery systems, cluster randomized trials (CRT) represent a key experimental design that may be used when individual randomization is not feasible due to administrative, financial or ethical reasons [1, 2].

Stepped-wedge designs (SWD) are a type of contemporary and novel CRT that have been used to evaluate new interventions and programs deployed in the context of routine implementation [3, 4]. A SWD is unique in that it combines key elements of cluster-randomized designs with a crossover component commonly used in longitudinal designs. Specifically, in SWD, all clusters (typically) start in the control group, cross over to the intervention group at different time points, and stay on intervention until the end of the trial. The time at which each cluster starts the intervention is randomized. Either different individuals (cross-sectional design) from each cluster may be measured at different time points or the same individuals may be repeatedly assessed (cohort design).

In the past 10 years, an increasing number of studies have used the SWD in health-related research within a broad range of domains, including HIV treatment, infection prevention, nutrition, asthma, cancer, and trauma. Recent SWD trials have been conducted in various global settings including America, Europe, Africa, Asia, and Australia. Compared to a standard parallel design CRT, the SWD is preferred in some circumstances due to practical, ethical, or methodological concerns [5–7].

Linear mixed models and generalized linear mixed models [8] are commonly used for the analysis of SWD data. A linear mixed model (LMM) is a type of regression model that includes random effects in addition to the standard fixed effects used in a linear model to account for dependence among observations from the same cluster. The use of random effects is a natural way to represent the heterogeneity among clusters under study and these methods produce valid inference when assumptions are satisfied. Generalized linear mixed models (GLMM) extend the LMM framework to non-normal data and non-identity links such as logistic regression for binary outcomes or Poisson regression for count data.

The increasing adoption of the SWD necessitates the development of flexible and valid sample size calculations. Hussey and Hughes [9] provided analytical formulae for power calculations based on repeated cross-sectional samples using a weighted least squares approach. The Hussey and Hughes [9] power calculations were based on a linear mixed model with a random cluster effect only. Woertman et al. [10] proposed a design effect that accounts for the inflation caused by the within-cluster correlation based on the Hussey and Hughes [9] formulation. Hooper et al. [11] reviewed designs for cluster randomized trials with repeated cross-sections and included random effects for time within clusters for stepped wedge designs. Hooper et al. [12] introduced sample size calculation for longitudinal CRTs including SWD, in which they include random effects for time within clusters for closed cohort designs. Hemming and Taljaard [13] summarized the design effects for SWD and CRT and provided a unified approach for their sample size calculation. Power calculations based on mixed models with random intervention effects may also be important to consider [14].

As an alternative to using the analytical expressions based on weighted least squares or maximum likelihood, Baio et al. [15] proposed simulation-based power calculations. The strategy is to specify a complete model that represents the data generating procedure with flexible choices for random effects and then calculate power using data generated by the model coupled with the planned primary analysis strategy. Simulation methods are computationally intense yet totally flexible and may be used with both cross-sectional and cohort study designs.

However, little research has been done on power calculation for non-normal responses such as binary and count outcomes when these are modeled on their natural regression scale such as logit or log, respectively. Rather, such data are commonly treated using linear model methods for the SWD which implicitly is either moment-based or assumes approximately normally distributed data. As a result, SWD power calculations for binary data are typically conducted in terms of risk differences or rate differences [11, 13]. However, it may be preferable to model the outcomes on the natural and unconstrained scale of interest particularly for the adoption of flexible multilevel models which can characterize multiple sources of heterogeneity. In the presence of fixed time effects (which are considered necessary for SWDs) or other fixed effects, contrasts such as risk differences generally cannot be translated to simple overall odds ratios associated with intervention due to the change of model scale. Existing methods for non-normal outcomes are limited to simulation-based power calculation strategies [15] and the exact maximum likelihood-based power calculation strategy [16]. Both of these approaches are computationally intensive and inhibit the exploration of a wide range of design configurations for a proposed study.

When the outcome is non-normal, a full maximum likelihood analysis for a GLMM based on the marginal distribution of the outcome in the observed data requires numerical or stochastic integration for the calculation of the log-likelihood. Breslow and Clayton [17] proposed a rigorous approximate inference method based on penalized quasi-likelihood (PQL). Dang et al. [18] use PQL approximations to propose sample size and power calculation based on GLMM with correlated binary outcomes. Similarly, Kapur et al. [19] considered sample size determination for longitudinal designs with binary response data using a two-level mixed effect logistic regression model. Amatya and Bhaumik [20] proposed a general methodology for sample size determination with hierarchical designs and their approach involves complex expressions that have to be solved iteratively using estimates of variance components. To facilitate the use of PQL-based sample size calculation in SWDs with non-normal outcomes, we propose a sample size and power calculation formula for SWDs with normal or non-normal outcomes by simplifying the Laplace approximation of the covariance matrix of the estimated parameter of interest. The method is intuitive, requires minimal computational power, and allows for rapid dynamic updates of sample size calculations when different parameters or design options are of interest. To facilitate adoption of our methods, we also provide a function embedded in the R package “swCRTdesign” [21] for sample size and power calculation for multilevel stepped wedge designs.

This paper is structured as follows. In “Method” section, we introduce our proposed method and provide an analytical formula for power/sample size calculation. In “Results” section, we use simulation experiments to compare the variance and power calculated by the proposed method with those given by the computationally intensive MLE-based method through repeated simulations. In “Discussion” section, we apply the proposed method to two studies, a public health patient-delivered partner therapy study for STI treatment and prevention with one level of clustering, and a health care delivery study with two levels of clustering that assesses the impact of providing additional benchmark prevalence information with a spine imaging report. Finally, we discuss the scope of application of the proposed method in “Appendix” section.

## Method

Denote the outcome for *n*_*j*_ observations from a given cluster *j* as $\boldsymbol {Y}^{n_{j}\times 1}_{j}=({Y}_{1j},\dots,{Y}_{n_{j}j})$, the design matrix for fixed effects as $\boldsymbol {X}^{n_{j}\times p}_{j}$, the coefficient vector for the fixed effects is denoted as ***β***, random effects as $\boldsymbol {b}^{q\times 1}_{j}$ with a design matrix $\boldsymbol {Z}^{n_{j}\times q}_{j}$, and *g*=*h*^−1^ as the link function. Suppose the mean and variance of the outcome take the following flexible GLMM form:

$$E\left[\boldsymbol{Y}_{j} \mid \boldsymbol{b}_{j}\right]\equiv \boldsymbol{\mu}_{j}^{\boldsymbol{b}_{j}}=h\left(\boldsymbol{X}_{j}\boldsymbol{\beta}+\boldsymbol{Z}_{j}\boldsymbol{b}_{j}\right)\equiv h\left(\boldsymbol{\eta}_{j}^{\boldsymbol{b}_{j}}\right), $$

1 $$ Var[Y_{{ij}}\mid \boldsymbol{b}_{j}]=\phi a_{{ij}} v({\mu}_{{ij}}^{\boldsymbol{b}_{j}}),   $$

where ***b***_*j*_∼*Normal*(0,***D***),*ϕ* is a dispersion parameter, *a*_*ij*_ is a known constant for each observation, and *v*() is a variance function. *ϕ*,*a*_*ij*_, and *v*() depend on the distribution of ***Y***_*j*_ (see Table 1). The outcomes are conditionally independent given the random effects. We will assume that the *p*^*th*^ column of ***X***_*j*_ corresponds to the intervention effect. Thus, the parameter of interest is *β*_*p*_.

**Table 1:** Variance function values for selected distributions and links ([22])

	*ϕ*	*a*	*v*(*μ*)	*g*(*μ*)	*g*^′^(*μ*)
Normal	*σ*^2^	1	1	*μ*	1
Bernoulli	1	1	*μ*(1−*μ*)	$log(\frac {\mu }{1-\mu })$	$\frac {1}{\mu (1-\mu)}$
Poisson	1	$\frac {1}{m_{i}}$	*μ*	*log*(*μ*)	$\frac {1}{\mu }$
Binomial	1	$\frac {1}{m_{i}}$	*μ*(1−*μ*)	$log(\frac {\mu }{1-\mu })$	$\frac {1}{\mu (1-\mu)}$

^*^The $\frac {1}{m_{i}}$ indicates that the *i*^*th*^ count is based on *m*_*i*_ intervals or units; typically, *m*_*i*_=1.

Typically, in a stepped-wedge design, the fixed effects consist of (at least) fixed time effect(s) and a fixed intervention effect. Random effects may consist of a random intercept, a random time effect(s), and/or a random intervention effect [14]. Additional random effects may be included in a cohort design to further characterize repeated measures on individuals within a cluster.

### Variance approximation

Breslow and Clayton [17] use Laplace’s method of integral approximation for marginalizing over the random effects in (1) to approximate the covariance matrix of the estimated parameter $\hat {\beta }$ by:

2 $$ Var(\hat{\beta})=\left(\boldsymbol{X}^{T}\boldsymbol{V}^{-1}\boldsymbol{X}\right)^{-1},   $$

where

$$\boldsymbol{V}=\boldsymbol{W}^{\boldsymbol{b}}+\boldsymbol{Z}\boldsymbol{D}\boldsymbol{Z}^{T}, $$

and ***W***^***b***^ denotes a diagonal matrix with entries $w^{\boldsymbol {b}}_{i}=\phi a_{i} v\left (\mu _{i}^{\boldsymbol {b}}\right)\left [g^{\prime }\left (\mu _{i}^{\boldsymbol {b}}\right)\right ]^{2}$, which depend on random effects ***b***. Here ***X*** and ***Z*** are design matrices for the fixed effect and the random effect for all observations across clusters. Breslow and Clayton [17] refer to their procedure as penalized quasi-likelihood or PQL. Note that PQL is an estimation strategy that focuses primarily on the regression parameters and the variance components; however, as part of the overall PQL approximation, individual random effects estimates, ***b***, are also available. To simplify the power calculation procedure with specified regression and variance component parameters, we propose the use of (2) with ***b*** set to their prior mean/mode of 0, that is, setting ***W***^***b***^=***W***^***0***^.

For cluster designs, assuming clusters are independent, (2) may be rewritten as $Var(\hat {\beta })=\left (\sum _{j}\boldsymbol {X}_{j}^{T}\boldsymbol {V}_{j}^{-1}\boldsymbol {X}_{j}\right)^{-1}$, where *j* is the index for cluster, and ***X***_*j*_ is a *n*_*j*_×*p* design matrix for cluster *j* with *n*_*j*_ observations. The terms on the right-hand side of (2) are computed separately for each cluster and the sum is over clusters. Our proposed variance estimator is theoretically well-justified ([17]) but relies on the essential PQL approximation and the plug-in value for random effects. The PQL approximation is exact when the outcome is normal with an identity link. For non-linear outcomes that we consider, extensive simulation evaluation is conducted below to detail operating properties of this strategy.

### Sample size and power calculation

For stepped-wedge clustered designs, the sample size is a combination of the number of clusters, the number of sequences, the number of time periods, and the number of individuals per cluster period. Power can be calculated given the sample size, or sample size may be computed given power by satisfying the equation ([23]):

3 $$ Power=\Phi\left(\frac{|\beta_{p}|-Z_{1-\frac{\alpha}{2}}\sqrt{V_{0}(\hat{\beta}_{p})}}{\sqrt{V_{a}(\hat{\beta}_{p})}}\right),  $$

where *α* is the (two-tailed) significance level, *β*_*p*_ is the intervention effect under the alternative hypothesis, and $V_{0}\left (\hat {\beta }_{p}\right)$ and $V_{a}\left (\hat {\beta }_{p}\right)$ are the variances of the estimated parameter under the null and alternative hypotheses, respectively.

## Results

### Simulation

In this section we use simulation experiments to compare the variance and power calculated by the proposed method with those given by simulation-based variance and power computations.

#### Simulation settings

We simulate binary outcomes with a Bernoulli distribution and a logit link since this scenario is biomedically important and known to be a situation for which PQL estimation may not perform well. We use a standard cross-sectional SWD in which the number of sequences is one less than the total number of time points. Specifically, we generate data from a SWD with four time periods and three sequences. For simplicity, each treatment sequence is set to have the same number of clusters (see below), and each cluster has the same number of individuals. The methodology can be applied to general settings with unequal number of clusters/individuals. We consider two outcome models with different random effects. Outcome Model I consists of a random intercept and a random effect for the treatment at the cluster level. Outcome Model II consists of a random intercept and a random time effect at the cluster level. Fixed effects include time effects and the treatment effect. Denote the number of time periods as *N*_*t*_. For cluster *j*, individual *i*, the data generating model takes the following form:

$$logit\left(Pr\left[{Y}_{{ij}}=1\mid \boldsymbol{b}_{j}\right]\right)=\boldsymbol{X}_{{ij}}\boldsymbol{\beta}+\boldsymbol{Z}_{{ij}}\boldsymbol{b}_{j}, $$

where

$$\boldsymbol{X}_{{ij}}=\left(\boldsymbol{1} \; \boldsymbol{X}_{time,j} \; X_{treatment,j}\right), \boldsymbol{\beta}=\left(\beta_{0} \; \boldsymbol{\beta}_{{time}} \; \beta_{p}\right), $$

where *X*_*time,j*_ is a *N*_*t*_−1 vector reparametrizing time as dummy variables. For Outcome Model I, ***Z***_*ij*_=(***1*** *X*_*treatment,j*_),***b***_*j*_=(*b*_*cluster,j*_,*b*_*treatment,j*_)∼*Normal*(***0***,***D***_1_),***D***_1_ is a diagonal matrix with elements $(\sigma _{{cluster}}^{2},\sigma _{{treatment}}^{2})$, assuming the two random effects are uncorrelated (the proposed method could also be implemented with correlated random effects). Similarly for Outcome Model II, $\boldsymbol {Z}_{{ij}}=\left (\boldsymbol {1} \; \boldsymbol {X}_{time,j}^{*}\right), \boldsymbol {b}_{j}=\left (b_{cluster,j},\boldsymbol {b}_{time,j}\right)\sim Normal\left (\boldsymbol {0},\boldsymbol {D}_{2}\right)$, where $\boldsymbol {X}_{time,j}^{*}$ is a *N*_*t*_ vector whose *n*^*th*^ elements is 1 and the rest of the elements are 0 where *n*_*t*_ is the time of observation for individual *i*, ***b***_*time,j*_ is a vector of length *N*_*t*_, and ***D***_2_ is a diagonal matrix with elements $\left (\sigma _{{cluster}}^{2},\boldsymbol {\sigma }_{{time}}^{2}\right)$, assuming the two random effects are uncorrelated.

Each simulated dataset is analyzed using a mixed model regression to generate maximum likelihood (ML) estimates (using function glmer() from “lme4” package). We compute the variance of the ML estimates over many simulations and compare this to the variance predicted by Eq. (2). We compute the ML-based power by simulating data under the alternative hypothesis and calculating the frequency of rejection, then compare it to the power computed using the predicted variance.

These comparisons are made under a range of scenarios, including ones for which the PQL approximation may perform poorly such as a small number of clusters, a high variance for the random treatment effect, small sample size within each cluster, and low prevalence.

Specifically, we consider 4, 8, or 12 clusters per sequence (so 12, 24, or 36 clusters in total, respectively), a low (0.03), moderate (0.12), or high (0.43) prevalence in the null effect group (log odds approximately equal to −3.5,−2.0, and −0.28, respectively), a cluster size of 20, 50, or 100, and an effect size of 0.2 (log odds ratio). On the log odds scale, the standard deviation of the random cluster effect is set to 0.05 and the standard deviation of the random treatment and time effects is set to be 0.05 or 0.1. The exact scenarios are given in Table 2.

**Table 2:** Parameter setting for binomial outcomes (log odds scale)

	Simulation	EPT Trial	LIRE Trial
# of sequence	3	4	5
# of time period (*N*_*t*_)	4	5	6
# of cluster per sequence	4, 8, 12	6	20
# of pcp per cluster	NA	NA	35
Cluster size	20, 50, 100	?	?
Prevalence (roughly)	0.03, 0.12, 0.43	0.08	0.19
Fixed time effect *β*_*time*_	(0.1, 0.1, 0.1)	(−0.008, −0.08, -0.17, −0.11)	−0.124
Effect size for the intervention *β*_*p*_	0.2	−0.3	−0.055
SD of random cluster effect	0.05	0.2	0.011
SD of random treatment effect	0.05, 0.1	NA	0.0054
SD of random time effect	0.05, 0.1	0.12	NA
SD of random cluster (pcp) effect	NA	NA	0.0015

^*^The cluster for LIRE Trial represents clinic, the random treatment effect is at clinic level, and time is modeled as a continuous variable

^**^SD stands for standard deviation

For each scenario, the variance of the estimated treatment effect coefficient $\hat {\beta }_{p}$ is computed across 2000 simulated datasets for the ML estimate and compared to Eq. (2) for the proposed method.

#### Simulation results

**Fig. 1:**
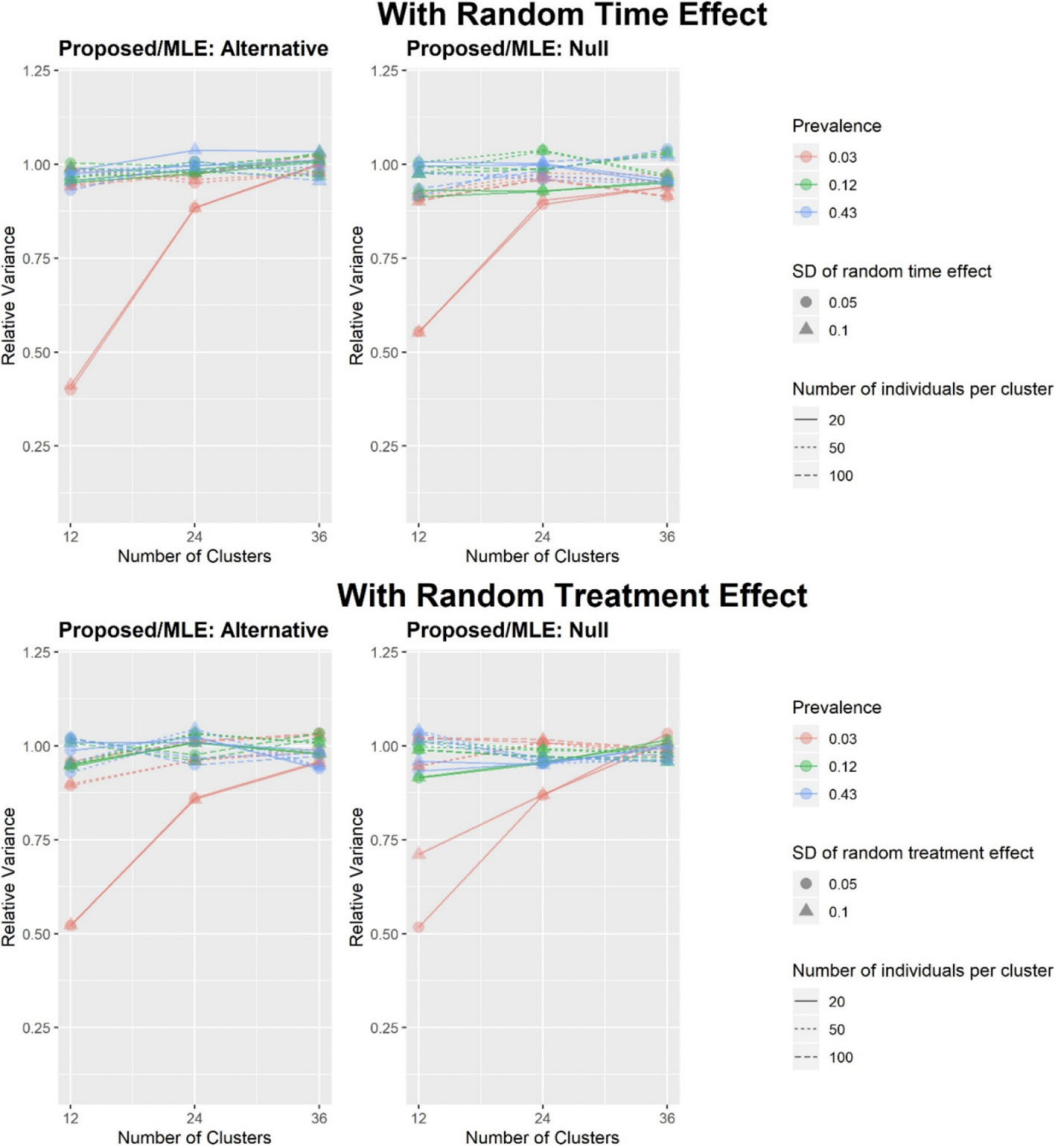
The relative estimated variance of $\hat {\beta }_{p}$ for the proposed method versus the MLE (variance measured across simulations), under both the alternative hypothesis (*β*_*p*_=0.2) and the null hypothesis (*β*_*p*_=0). The standard deviation of the treatment effect is on log odds scale

The relative variance of the treatment effect coefficient *β*_*p*_ for the proposed method compared to the MLE (as measured across simulations) is displayed in Fig. 1, under both the null and alternative hypotheses.

Figure 1 shows that in the presence of a random treatment effect, the relative variance between the ML-based method and the proposed method, under the alternative and the null hypotheses, is close to 1 in most scenarios except in the extreme case where the number of clusters is small (with a total of 12 independent clusters), the cluster size is small (with 20 subjects per cluster), and the prevalence is 0.03. In this extreme case, the variance estimate given by the proposed method is smaller than the actual variance of the MLE which could lead to an overestimate of power.

**Fig. 2:**
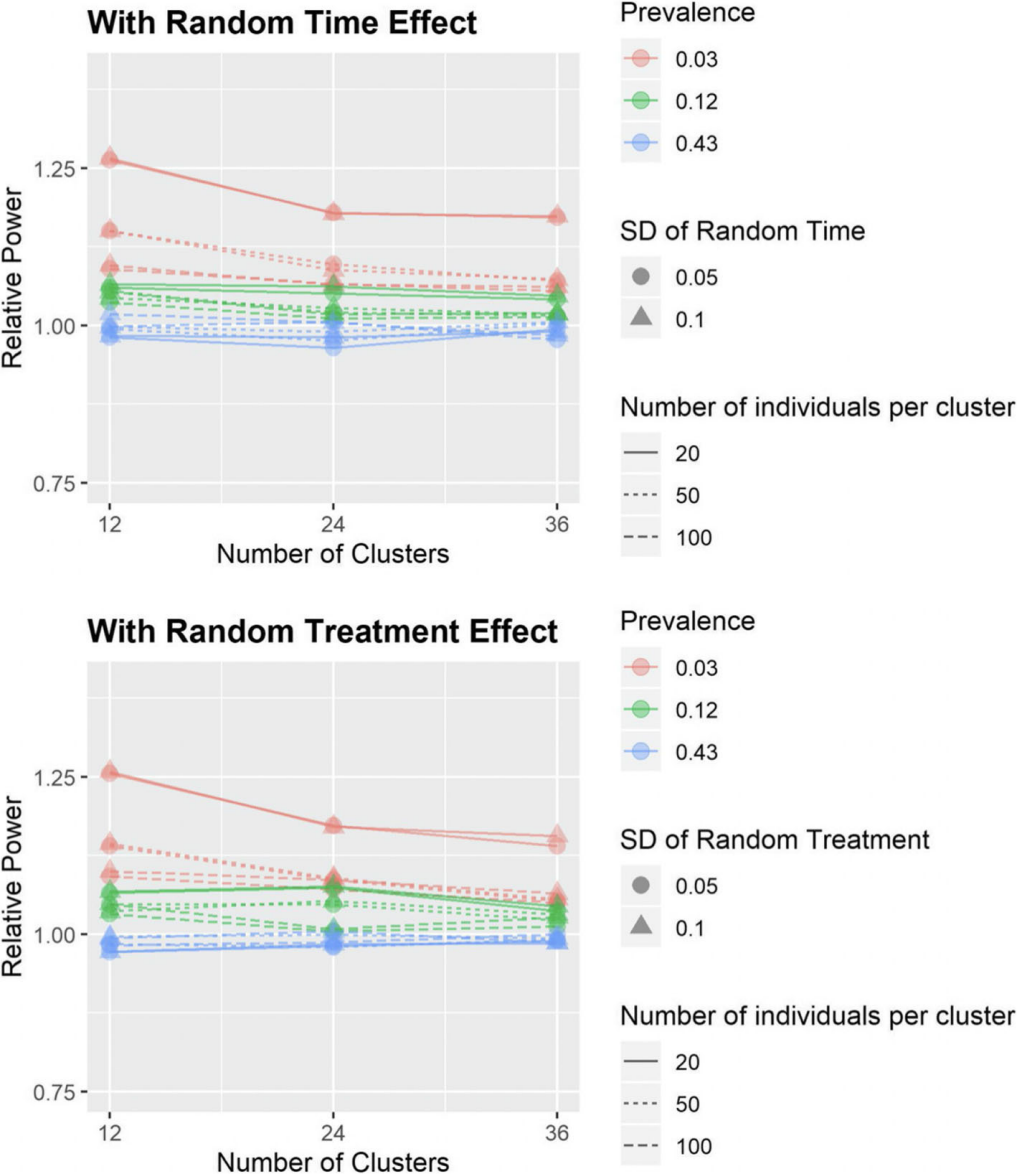
The relative estimated power (around 80%) of $\hat {\beta }_{p}$ for the proposed method versus the MLE (variance measured across simulations), under effect size of 0.2 (log odds ratio)

Relative estimates of power, comparing the proposed method and the MLE for situations when the power according to MLE is around 80% (achieved by varying the effect size), are displayed in Fig. 2. Here we target comparison at the common benchmark power of 80%. A relative power close to 1 is favorable for it indicates that the proposed method does not over- or under-estimate the power.

Note that the relative variance plots (Fig. 1) are not directly comparable to the relative power plot (Fig. 2) because the effect sizes (all greater than 1) in the latter are chosen such that the estimated power is approximately 80% for the MLE. As a result, the prevalence may not be low under the alternative hypothesis. Therefore, the poor performance of the proposed method in the extreme scenarios in Fig. 1 does not carry over to Fig. 2.

Figure 2 shows that when the estimated power calculated by MLE is around 80%, in the presence of random treatment effect or random time effect, the relative power between the ML-based method and the proposed method is close to 1 implying validity for use in sample size and power planning exercises.

### Applications

#### Application with a simple data structure: partner notification

Patient delivered partner therapy (PDPT) is a partner notification strategy for individuals with sexually transmitted infections (STIs). Drugs or drug vouchers are given to patients with STIs to give to their sex partners.

The effectiveness of a PDPT-based partner notification strategy dubbed EPT (expedited partner therapy) was established by an individually randomized trial conducted in King County, Washington, between 1998 and 2003 for chlamydia and/or gonorrhea infection treatment.

EPT was then implemented in all counties in Washington between 2007 and 2009 through a cluster randomized trial using a stepped wedge design. County-based health districts in Washington state were randomized to EPT at one of four possible time periods with 5–6 districts at a time. Each time interval was 6–8 months. The prevalence of chlamydia was measured using cross-sectional sampling among women tested in family planning clinics for each county in each time interval.

The proposed model (see appendix 6.1) includes random cluster and time effects and we use the coefficient estimates from the final analysis of chlamydia to demonstrate the use of the proposed method in sample size calculation (Table 2). With an effect size of −0.3 (log odds ratio), prevalence of chlamydia must be measured in approximately 140 women in each cluster period in 24 counties to achieve a power of 80%.

#### Application with a complex data structure: Lumbar Imaging with Reporting of Epidemiology (LIRE)

Incidental anatomic spine findings given by diagnostic imaging may lead to unnecessary additional tests and treatments among pain-free individuals. However, research has suggested that primary care patients were less likely to receive subsequent tests or medical interventions if the radiology report provides addition information on the prevalence of imaging finding among patients without back pain. Thus, Roland and van Tulder [24] proposed providing reference prevalence of various degenerative findings among patients without back pain in the spine imaging report to help reduce unnecessary medical attention.

A large, prospective stepped wedge cluster randomized control trial was designed to assess the impact of providing additional benchmark prevalence information in the imaging report.

A total of 100 primary care clinics from four large health systems were randomized to initiate the intervention at one of five possible times, each 6 months apart. The number of sequences, time periods, and the number of clusters per sequence are given by the study protocol ([25]). The randomization was stratified by the clinic size. The size of the clinic was determined by the number of primary care providers (PCPs) and the site of the clinic. We examine the power calculation for the secondary outcome, the indicator of any opioid prescription within 90 days of the index imaging study. The random effect structure considered in our power calculation parallels those considered in the original power calculation for the primary outcome. The effect sizes and standard deviations of random effects for the power calculations we present are given by a model fit to the actual data from the trial (Table 2). The model is included in appendix 6.2.

**Fig. 3:**
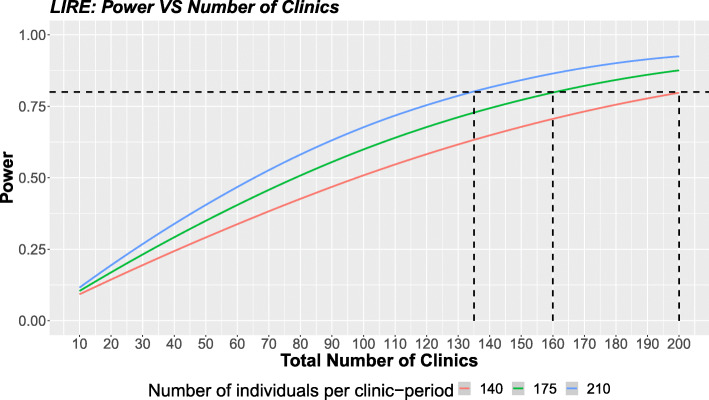
The relationship between the total number of clinics and the power with different number of individuals per clinic-period

We consider power calculation for LIRE to demonstrate the use of the proposed method for a problem with two-levels of clustering (clinic level and PCP level), which is computationally intensive for simulation-based methods and effectively impossible for existing exact methods. With an effect size of −0.055 (log odds ratio), Fig. 3 shows the relationship between the total number of clinics and the power. When outcomes are measured in 140, 175, or 210 patients per clinic-period, approximately 135, 160, and 200 clinics are needed to achieve a power of 80%. Figure 3 shows the value of high-fidelity approximation methods since we can explore a wide range of design alternatives with a computationally feasible strategy. To illustrate the difference in computing burden between our method and the simulation-based method, we have calculated the time required for the two methods to explore the 60 different scenarios presented in Fig. 3 for the LIRE trial. The results are included in appendix 6.3.

## Discussion

Power/sample size calculations are difficult for clustered data when the outcome is non-normal. Specifically, use of a normal approximation may be poor and there are no analytical formulae for power under non-identity links. For stepped-wedge designs, the assumed time trend can affect power when the outcome model has a nonlinear link. Existing methods for non-normal outcomes, including simulation-based or exact ML-based power calculations, are computationally intensive.

In this paper, we propose a sample size and power calculation formulae for SWD with normal or non-normal outcomes using a Laplace approximation of the covariance matrix of the estimated parameter of interest. The method is fast computationally and has a good performance under non-extreme cases with a reasonable number of clusters, cluster sizes, and prevalence. This approach can be extended to any design as long as the outcome model can be specified in the form of a generalized linear mixed model (model 1), which includes cohort designs, incomplete designs, or exponential decay models [26].

By allowing one to compute power on the same scale as the intended analysis, the proposed approach can provide a more accurate estimate of study power. In addition, boundary issues (i.e., proportions outside the range 0–1) are avoided by working on canonical scales such as the logit or log. Of course, this also means that the variances of random effects must be specified on those same scales even though, at present, most published values for variance components are given for an identity link scale. Also, as noted previously, the assumed time trend may affect power when computed on nonlinear scales so greater attention must be given to this component during the design phase.

Implementing the proposed method involves taking the inverse of matrices *V*_*j*_’s, which has dimension equal to the cluster sizes. This can take extended computing time with large clusters. One way to speed up the inversion is to use the Woodbury matrix identity [27], which requires inversion of a matrix of size equal to the dimension of the random effect vector *b*_*j*_ instead. As long as the dimension of *b*_*j*_ is smaller than the cluster size, the inversion is faster.

The extreme cases where the variance given by the proposed method does not reflect the actual cross-simulation variance of the MLE are characterized by settings with a combination of a small number of clusters, small sample sizes, and extreme prevalence. Indeed, in simulations, we also find that the mean of the variance estimates from ML estimation often does not match the true cross-simulation variance in these situations. These are likely to be cases in which other approximate power calculations (such as the existing closed-form formulas that approximate non-normal outcomes using the normal distribution) also perform poorly [16].

In summary, the proposed method provides a unified procedure for sample size calculations for stepped wedge design trials based on linear and generalized linear mixed models. The method is computationally fast and so allows for easy exploration of a variety of designs and parameter values. To make the greatest use of these methods, it is important that researchers reporting results from completed trials publish variance component values on the natural analytic scales (e.g., logit and log) for binary and count data.

## Appendix

### Model for the EPT Trial

$cluster: j=1,\dots,J; individual: i = 1, \dots, I; time: t=1,\dots, T$$E(\boldsymbol {Y}_{j}\mid \boldsymbol {b}_{k})=h(\boldsymbol {X}_{j}\boldsymbol {\beta }+\boldsymbol {Z}_{j}\boldsymbol {b}_{j}), \boldsymbol {\beta }=(\beta _{0},\beta _{{time}},\beta _{{tx}}), \boldsymbol {b}_{j}=(b_{j,0},b_{1j},\dots,b_{{Tj}}), \boldsymbol {Y}_{j}=(Y_{1j},\dots,Y_{{Ij}})$,$\boldsymbol {X}_{j}=(\boldsymbol {1}_{I},(\boldsymbol {time}_{1j},\dots,\boldsymbol {time}_{{Ij}})^{T},(tx_{1j},\dots,tx_{{Ij}})^{T}), \boldsymbol {time}$ are (*T*−1) vectors with 1 at exactly 1 place (reparametrizing time as dummy variables, -1 degrees of freedom). ***Z***_*j*_=(***1***_*I*_,***A***),***A*** is a *TI*×*T* matrix consists of time dummy variables.

### Model for the LIRE Trial

$clinic: k=1,\dots,K; pcp: j=1,\dots, J; individual: i = 1, \dots, I; time: t=1,\dots, T$$E(\boldsymbol {Y}_{j}\mid \boldsymbol {b}_{k})=h(\boldsymbol {X}_{j}\boldsymbol {\beta }+\boldsymbol {Z}_{j}\boldsymbol {b}_{j}), \boldsymbol {\beta }=(\beta _{0},\beta _{{time}},\beta _{{tx}}), \boldsymbol {b}_{k}=(b_{k,0},b_{k,1},b_{1k,0},\dots, b_{Jk,0})$,$ \boldsymbol {Y}_{k}=\!(Y_{11k},Y_{21k},\dots,Y_{I1k},Y_{12k},\dots,\!Y_{I1k},\dots,Y_{1Jk},\dots,\!Y_{{IJk}})$,$\boldsymbol {X}_{k}=(\boldsymbol {1}_{{IJ}}, (time_{11k},time_{21k},\dots,time_{I1k},\dots, time_{1Jk},\dots, time_{{IJk}})^{T}$,$(tx_{11k},tx_{21k},\dots,tx_{I1k},\dots, tx_{1Jk},\dots, tx_{{IJk}})^{T})$,$\boldsymbol {Z}_{k}=(\boldsymbol {1}_{{IJ}}, (tx_{11k},tx_{21k},\dots,tx_{I1k},\dots, tx_{1Jk},\dots, tx_{{IJk}})^{T},\boldsymbol {B}), \boldsymbol {B}=diag(\boldsymbol {1}_{I}, \dots, \boldsymbol {1}_{I})_{IJ\times J}$.

### Computation time comparison

To better illustrate the difference in computing burden between our method and the simulation-based method, we have calculated the time required for the two methods to explore the 60 different scenarios presented in Fig. 3 for the LIRE trial in which multilevel clustering is present for non-normal outcomes (see Table 3).

**Table 3:** Calculation time for the 60 scenarios considered in the paper for the LIRE trial

Number of individuals per clinic-period	Methods	Computing time (in days)
140	Analytic	0.02
	Simulation-based	12.43
175	Analytic	0.04
	Simulation-based	16.11
210	Analytic	0.07
	Simulation-based	19.88

To summarize, the table compares the calculation time for the simulation-based method with 1000 replicates and the proposed analytic method for a total of 60 scenarios using a single core of a 2.6 GHz Intel Core i7 processor. As expected, the simulation-based method’s calculation time increases drastically as the number of individuals considered increases, but the calculation time of the analytic method is consistently short. The table shows the total number of days taken for both methods to explore the 60 scenarios. It takes weeks for a simulation-based method to explore all scenarios, but the analytic method takes less than 2 h. In fact, exploring just one single scenario using the simulation-based method can take up to 2 days in this example.

Moreover, since the simulation-based methods rely on replicates that successfully converge without parameter estimates on the boundary, the actual time taken to get 1000 successful replicates could be much longer.

## Data Availability

Not applicable.

## Abbreviations

CRT: cluster randomized trials
SWD: stepped wedge designs
LMM: linear mixed models
GLMM: generalized linear mixed models
PQL: penalized quasi-likelihood
MLE: maximum likelihood estimation
PDPT: patient delivered partner therapy
STI: sexually transmitted infections
EPT: expedited partner therapy
LIRE: lumbar imaging with reporting of epidemiology
PCP: primary care providers
